# What single neurons can tell us

**DOI:** 10.7554/eLife.44560

**Published:** 2019-02-05

**Authors:** Elaine N Miller, Chet C Sherwood

**Affiliations:** 1 Department of AnthropologyThe George Washington UniversityWashingtonUnited States; 2 Center for the Advanced Study of Human PaleobiologyThe George Washington UniversityWashingtonUnited States

**Keywords:** human neurons, dendrites, pyramidal cells, intelligence, iq scores, human cortex, Human

## Abstract

IQ scores are correlated with the morphology and activity of certain neurons in the human temporal cortex.

**Related research article** Goriounova NA, Heyer DB, Wilbers R, Verhoog MB, Giugliano M, Verbist C, Obermayer J, Kerkhofs A, Smeding H, Verberne M, Idema S, Baayen JC, Pieneman AW, de Kock CP, Klein M, Mansvelder HD. 2018. Large and fast human pyramidal neurons associate with intelligence. *eLife* **7**:e41714. doi: 10.7554/eLife.41714

You probably remember being at school as a child and learning how to do arithmetic, to read and comprehend stories, and to solve puzzles. These tasks may appear simple to you now, but they are actually quite demanding because they require a high level of brain processing power. For decades, scientists have been working out ways to quantify our ability to take in knowledge and apply it to new situations – in other words, our intelligence. They have also explored the characteristics of the human brain that contribute to individual differences in performance on such tasks.

IQ tests are used to quantify intelligence by assessing an individual’s responses to timed questions in verbal comprehension, perceptual reasoning and working memory. Many hypotheses have been advanced to tie neural features to individual differences in test results. In humans, some studies have shown that total brain size is correlated with the level of intelligence; other work has revealed that intellect is related to having better connections between specific brain regions, such as the prefrontal and parietal cortices (McDaniel, 2005; Hearne et al., 2016). Furthermore, comparisons across mammalian species suggest that executive cognitive performance may correlate with brain size or with the sheer number of neurons in the cerebral cortex (MacLean et al., 2014; Herculano-Houzel, 2017). While these findings contribute to our understanding of how brains are built to make complex calculations, it had never been possible to test whether the fine structure of neurons is correlated with variation in human intellect.

Now, in eLife, Natalia Goriounova of the Vrije Universiteit Amsterdam and colleagues report that the microscopic anatomy of neurons and their physiological characteristics are linked to individual differences in IQ scores (Figure 1; Goriounova et al., 2018). The group had the remarkable opportunity to study samples of live temporal cortex removed during surgeries of cancer and epilepsy patients. Goriounova et al. examined certain characteristics of the neurons inside these tissues, and recorded their electrical activity. Then, they looked into whether these variables were associated with intelligence scores based on IQ assessment. Assigning a single value to one’s intellect has been controversial (Gould, 1981), but the team used IQ scores as a tractable measure that may reflect some aspects of cognitive information processing.

**Figure 1.**
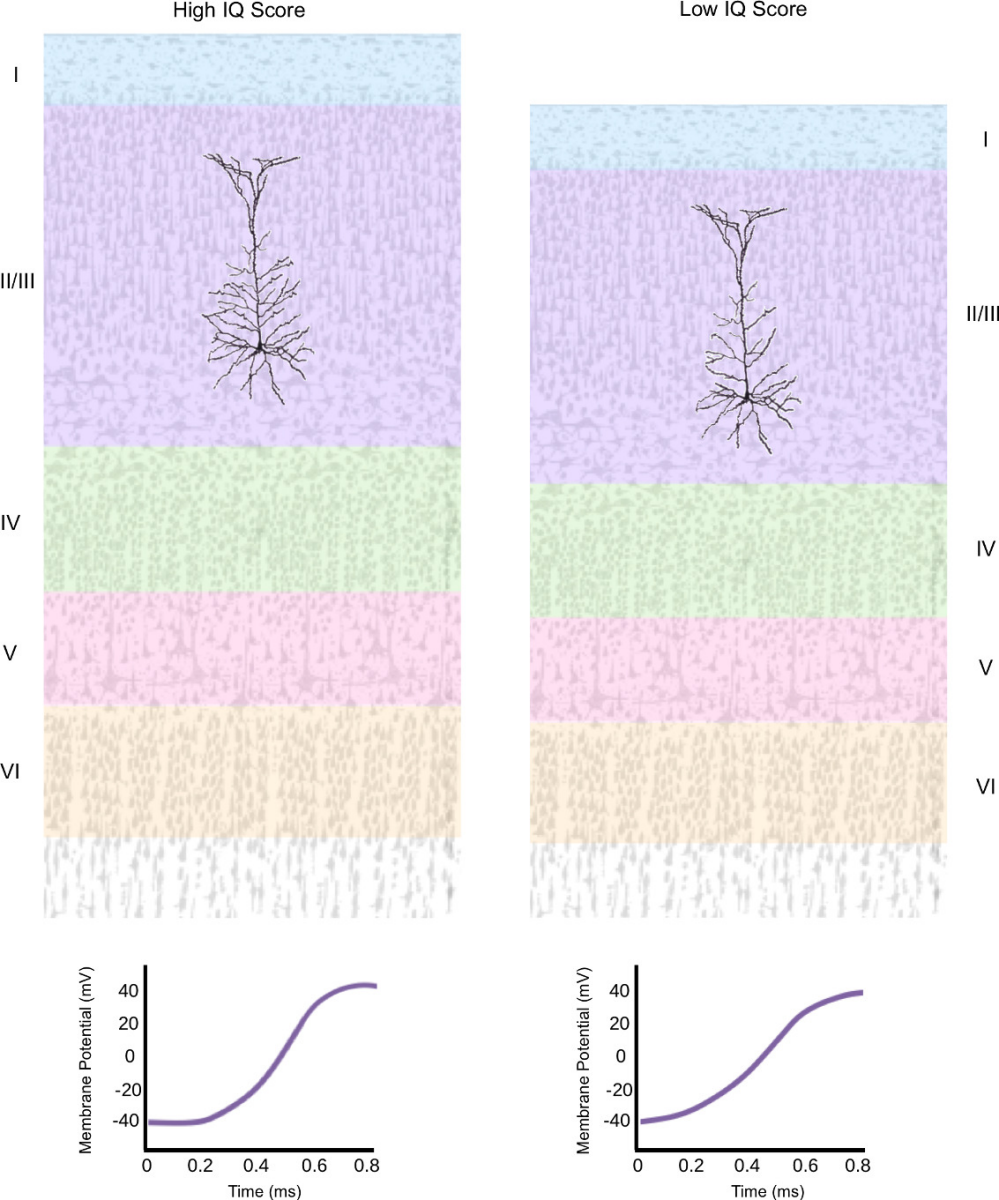
The architecture of individual neurons in the human cortex is connected to IQ scores. The temporal cortex in the human brain is organized in layers, which contain pyramidal neurons (black).These cells collect information from their neighbors through branch-like structures known as dendrites, and then integrate and transmit the message to other areas in the cortex. Goriounova et al. found that a higher IQ score (left) was associated with a thicker temporal cortex, which features pyramidal neurons with morethat have more elaborate dendritic networks and that fire faster (graph). A lower IQ score (right) was associated with a thinner temporal cortex which contains pyramidal neurons that have less complex dendritic networks and that fire slower.

First, the researchers, who are based at various institutes in Amsterdam, Zwolle and Antwerp, confirmed that higher IQ scores correlate with a thicker temporal cortex, based on measurements of pre-operative MRI scans. Then, for each patient, two or three pyramidal neurons from the upper cortical layers were selected and measured. These large cells are the principal type of neurons found in the cerebral cortex. They receive information from neighboring cells through dendrites, branch-like extensions that connect to other neurons at structures called synapses. The pyramidal neurons then ‘fire’ to transmit the message. Differences in dendritic length and branching explained approximately 25% of the variance in IQ scores between individuals in a sample of 25 patients. Longer dendrites have extra surface area, which could help increase the number of synapses the neuron can form. With more of these connections, the pyramidal neurons can produce an output signal that integrates more inputs from neighboring neurons in a given time.

Finally, computational models were used to explore how changes in the morphology of dendrites might influence the way the neurons worked. The analyses show that pyramidal neurons with larger dendritic trees fire more quickly, which allows them to transmit information faster. Indeed, recording the activity of cells in brain slices obtained from 31 patients demonstrated that higher IQ scores were associated with neurons firing more quickly, especially during sustained neuronal activity. Even this slight increase in how fast neurons pass on information can improve reaction times, and ultimately influence behavioral responses (Nemenman et al., 2008). With approximately 16 billion neurons in the human cerebral cortex, small differences in anatomical and physiological properties may alter intellectual performance (Herculano-Houzel, 2009).

Goriounova et al. have shown that distinct changes in the microanatomy of pyramidal neurons influence the working properties of these cells. Relatedly, a thicker cortex may arise from pyramidal cells with more elaborate dendritic networks. In turn, this would lead to more rapid information processing, and ultimately, increased intellectual performance. Similar observations have been made in one of our closest living relatives, the chimpanzees, where thicker cerebral cortices are associated with higher scores on intelligence tests (Hopkins et al., 2018). Human cortices also have more robust dendritic networks than chimpanzees, which may explain the differences in cognitive abilities between human and non-human apes (Bianchi et al., 2013).

However, characteristics at the level of the neuron explain only a minority of variation in IQ scores; other molecular, connectional or regulatory features may also play an essential role. Taken together, these results are helping us grasp the neural basis and evolution of human intelligence.
